# Ultrasound-Targeted Microbubble Destruction Enhances Inhibitory Effect of Apatinib on Angiogenesis in Triple Negative Breast Carcinoma Xenografts

**DOI:** 10.1155/2021/8837950

**Published:** 2021-04-17

**Authors:** Dengke Hong, Jiajia Yang, Jingjing Guo, Yu Zhang, Zhikui Chen

**Affiliations:** ^1^Department of Vascular Surgery, Fujian Medical University Union Hospital, Fuzhou, Fujian, China; ^2^Department of Ultrasound, Fujian Medical University Union Hospital, Fuzhou, Fujian, China

## Abstract

Ultrasound-targeted microbubble destruction (UTMD) has been proven as an effective technique to assist drugs to cross the vascular wall and cell membrane. This study was aimed at evaluating the synergistic antiangiogenic and growth-inhibiting effects of apatinib (APA) and UTMD on the triple negative breast cancer (TNBC). The TNBC xenograft model was established in nude mice (*n* = 40) which were then randomly divided into the APA plus UTMD (APA-U) group, UTMD group, APA group, and model control (M) group (*n* = 10 per group). Corresponding treatment was done once daily for 14 consecutive days. The general condition and body weight of tumor-bearing nude mice were monitored. Routine blood test and detection of liver and kidney function were done after treatments. The tumor size and microcirculation were examined by two-dimensional ultrasonography (2DUS) and contrast-enhanced ultrasonography (CEUS), respectively. Then, the tumor tissues were harvested for the detection of vascular endothelial growth factor (VEGF) by immunohistochemistry and for CD31-PAS double staining to assess microvessel density (MVD) and heterogeneous vascular positivity rate. After treatments, the tumor growth and angiogenesis were significantly inhibited in the APA group and the APA-U group, and these effects were more obvious in the APA-U group. The tumor volume, CEUS parameters, VEGF expression, and MVD in the APA-U group were significantly lower than those in the APA group (*P* < 0.05), while there were no marked differences in the heterogeneous vascular positivity rate, body weight, and blood parameters between the two groups (*P* > 0.05). In the UTMD group, the tumor growth and angiogenesis were not significantly inhibited, and all the parameters were similar to those in the M group (*P* > 0.05). During the experiment, all mice survived and generally had good condition. In conclusion, APA combined with UTMD may exert synergistic antiangiogenic and growth-inhibiting effects on the TNBC and not increase the heterogeneous vasculature and the severity of APA-related systemic side effects.

## 1. Introduction

The triple negative breast cancer (TNBC) is a special subtype of breast cancer which has no expression of estrogen receptor, progesterone receptor, and human epidermal growth factor receptor 2. TNBC is highly aggressive and easily recurs and migrates. Currently, there is no effective treatment for TNBC [1]. Angiogenesis is closely related to the growth and metastasis of malignant tumors, and antiangiogenesis has been accepted as a breakthrough in the treatment of malignant tumors [2]. The regulation of angiogenesis mainly depends on the interaction between vascular endothelial growth factor (VEGF) and vascular endothelial growth factor receptor (VEGFR) family members. Compared with other subtypes of breast cancer, TNBC has significantly higher expression of VEGF and VEGFR, which is closely related to the poor prognosis of TNBC. These support the application of antiangiogenic treatment for TNBC [3–5].

Currently, the common antiangiogenic drugs are VEGF inhibitors and VEGFR inhibitors, including bevacizumab, sorafenib, and sunitinib. However, clinical efficacy of these drugs has not been confirmed in clinical trials, and they usually have some side effects, which limit the wide clinical application of these drugs [6–10]. Apatinib (APA) is an oral small-molecule antiangiogenic drug and can selectively inhibit the tyrosine kinase activity of intracellular VEGFR2 and suppress the interaction between VEGF and VEGFR, exerting an antiangiogenic effect. Clinically, APA is mainly used as the third line treatment of advanced gastric cancer and gastroesophageal junction adenocarcinoma, and the efficacy and adverse reactions of APA in the treatment of TNBC are still being investigated in clinical trials [11–13].

Ultrasound-targeted microbubble destruction (UTMD) is an effective new technique that can assist drugs to cross the vascular wall and cell membrane [14]. This study was aimed at using APA combined with UTMD in the treatment of TNBC xenografts in nude mice. The body weight, routine blood parameters, tumor volume, blood perfusion, VEGF expression, and angiogenesis in the tumor tissues were assessed, with the aim to explore whether UTMD was able to enhance the antiangiogenic and growth-inhibiting effects of APA on the TNBC.

## 2. Materials and Methods

### 2.1. Reagents and Instruments

APA (Hengrui Medicine, Jiangsu, China), SonoVue (Bracco Imaging BV, Switzerland), fetal bovine serum (FBS) and L-15 medium (Gibco, USA), mouse anti-human VEGF monoclonal antibody (Maixin-Bio, Fujian, China), mouse anti-human CD31 monoclonal antibody (Maixin-Bio, Fujian, China), Aplio 500 ultrasound diagnostic system (Toshiba, Japan), and ultrasound therapy apparatus (Shengxiang, Shenzhen, China) were used in the present study.

### 2.2. Establishment of Animal Model

Forty BALB/cnu-nu female nude mice aged 5-6 weeks and weighing 16-20 g (SLACK, Shanghai, China) were housed in the specific pathogen-free (SPF) environment. Human breast cancer cell line MDA-MB-231 (Shanghai Cell Bank, Chinese Academy of Sciences) was maintained at 37°C in L-15 medium containing 10% FBS. When the cell confluence reached 100%, cells in the logarithmic growth phase were harvested, digested, and centrifuged, and then cells were resuspended in normal saline at a density of 2 × 10^7^/ml. Each mouse was subcutaneously inoculated with 0.1 ml of cell suspension at the right axillary.

### 2.3. Grouping and Treatment

Two-dimensional ultrasonography (2DUS) was done to monitor the tumor growth. When the average tumor diameter reached 5 mm, 40 tumor-bearing nude mice were randomly divided into four groups: APA plus UTMD (APA-U) group, UTMD group, APA group, and model control (M) group (*n* = 10 per group). APA was dissolved in 0.5% sodium carboxymethylcellulose (CMC-Na) at 7.5 mg/ml. Mice in the APA-U group and UTMD group were intragastrically administered with APA and 0.5% CMC-Na, respectively, at 10 ml/kg, and UTMD was done 2 h later; mice in the APA group and M group were intragastrically administered with APA and 0.5% CMC-Na, respectively, at 10 ml/kg without UTMD. UTMD was performed as follows: mice received an intravenous bolus of SonoVue at 5 ml/kg, and then the probe of an ultrasound diagnosis system was placed on the surface of the tumor, with thick gel to remove the gas between the probe and the tumor. The acoustic beam produced by the probe was cylindrical with a sectional area of 8.0 cm^2^, and ultrasound exposure was done with a power of 0.75 W/cm^2^ for 2 min (frequency, 840 kHz; focus depth, 1-2 cm; duration of irradiation, 10 s; interval, 10 s; a total of six cycles). Treatment was done once daily for 14 consecutive days.

### 2.4. General Condition and Body Weight Measurement

The diet, activity response, mental state, and skin condition of the nude mice were observed during the experiment, and the body weight was measured. The body weight curve was delineated, and the weight change rate was calculated as follows: weight change rate (%) = (weight before the first treatment − weight after the last treatment)/weight before the first treatment × 100%.

### 2.5. Measurement of Tumor Volume

Aplio 500 ultrasound diagnostic system equipped with PLT-805AT probe (central frequency, 9.0 MHz) was employed for 2DUS, and the maximum length (*L*), width (*W*), and height (*H*) of the tumors were measured every other day. The tumor volume (*V*) was calculated as follows: *V* = *πLWH*/6, and a growth curve was delineated.

### 2.6. Contrast-Enhanced Ultrasonography

Contrast-enhanced ultrasonography (CEUS) was performed before the first treatment and one day after the last treatment. After mice were intraperitoneally anesthetized with 2% lidocaine hydrochloride at 5 *μ*l/g, CEUS was performed with an Aplio 500 ultrasound diagnostic system, equipped with a PLT-805AT probe (central frequency, 9.0 MHz; mechanical index, 0.2), after fast bolus injection of SonoVue at 5 ml/kg through the tail vein. The raw data were recorded and further analysed using TCA software. The region of interest (ROI) was outlined in the tumor, the time intensity curve (TIC) was created, and the CEUS parameters including peak intensity (PI) and area under the curve (AUC) were measured to evaluate the tumor microcirculation.

### 2.7. Blood Test of Nude Mice

One day after the last treatment, the blood of the nude mice was collected by picking the eyeball and then anticoagulated with EDTA for cell sorting, including the white blood cell (WBC), platelet (PLT), and neutrophil (NE) ratios. Meanwhile, the serum was collected by centrifugation, and the levels of alanine aminotransferase (ALT) and creatinine (Scr) were determined for the assessments of the liver and kidney function.

### 2.8. Detection of VEGF Expression in Tumor Tissues

After blood collection, nude mice were sacrificed by intraperitoneal injection of 2% sodium pentobarbital at 100 mg/kg. Then, the tumor tissues were harvested and fixed in 10% formaldehyde, embedded in paraffin, sectioned, and then processed for immunohistochemistry for VEGF. PBS was used as a negative control instead of the primary antibody, and a known positive section was used as a positive control. The presence of brown granules in the cytoplasm was considered positive to VEGF expression. Five high-magnification fields (×400) were selected randomly from each section under a light microscope, and representative figures were captured and semiquantitatively analysed with ImageJ software. The integrated optical density (IOD) was measured as the expression of VEGF in the target area, and the average IOD of 5 fields was calculated for final analysis.

### 2.9. Detection of Neovascularization in Tumor Tissues

The tumor sections were processed for CD31-PAS double staining, and PBS served as a negative control instead of the primary antibody, while the positive section was used as a positive control. The new blood vessels in the tumor tissues were observed under a light microscope, including microvessel (MV) and heterogeneous vasculature. The presence of cytoplastic brown granules was regarded as positive staining for vascular endothelial cells. The tumor cells with positive CD31 expression should be distinguished carefully: a single endothelial cell, endothelial cell cluster, or vascular structure with a clear boundary from the surrounding structure was regarded as one MV, and blood vessels with obvious muscle layer and blood vessels with a long diameter greater than 50 *μ*m were excluded. Five high-magnification fields (×400) were selected randomly for each section, and the MVs were counted, followed by calculation of the average as the microvessel density (MVD). The heterogeneous vasculature included mosaic blood vessel and vascular mimicry (VM). The mosaic blood vessel was composed of endothelial cells and tumor cells without red blood cell and inflammatory cell infiltration around it. In the present study, CD31-PAS double staining was used to identify VM [15, 16]. VM was characterized by a lumen-like structure surrounded by tumor cells, with no endothelial cells and negative expression of CD31, and the basement membrane of the VM could be positive to PAS or purple-red, with red blood cells in the lumen. When the positive staining in the section was weak and difficult to judge, ImageJ software was used for assistant analysis.

### 2.10. Statistical Analysis

The SPSS version 24.0 software package was used for statistical analysis. The quantitative data are expressed as mean ± standard deviation ($\bar{x}\pm \text{SD}$). The body weight, weight change rate, tumor volume, CEUS parameters, blood parameters, VEGF expression, and MVD among groups were compared with one-way analysis of variance (ANOVA) followed by least significant difference *t*-test (LSD *t*-test). When the heterogeneity of variance was observed, the Tamhane T2 test was used. The total and positive heterogeneous vasculatures in each group were counted, followed by the calculation of the positive rate. Fisher's exact test was used to compare the heterogeneous vascular positivity rate among groups, and the paired comparisons were performed by chi-square test, using the Bonferroni method to adjust the test level to 0.0125. A value of *P* < 0.05 was considered statistically significant.

## 3. Results

### 3.1. Body Weight and General Condition of Nude Mice

**Figure 1:**
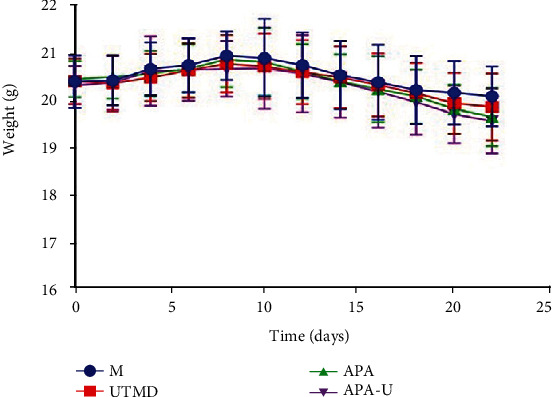
The body weight curve of nude mice in different groups.

During the experiment, all the nude mice in each group had good diet, activity response, and mental state, and their skin condition was normal, without redness and rash. During the experiment, the body weight in each group increased slowly at first and then decreased slightly. At the end of the experiment, the body weight in each group decreased slightly compared with that before treatments and before the inoculation, but there were no significant differences in the body weight and weight change rate among different groups (*P* > 0.05) (Table 1). The body weight curve of nude mice in different groups is shown in Figure 1.

At the beginning of the experiment, the body weight in each group increased slowly. On the 8^th^ day after the inoculation, the body weight in the M group, UTMD group, and APA group began to decline slowly, while in the APA-U group, it began to decline slowly on the 10^th^ day after the inoculation.

### 3.2. Tumor Volume

**Figure 2:**
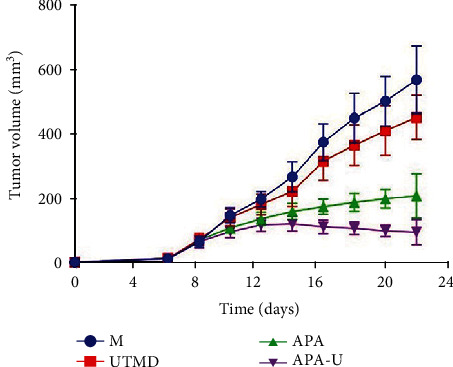
The tumor growth curve in different groups.

On the 8^th^ day after tumor inoculation, the mean diameter of the subcutaneous transplanted tumor measured by ultrasound was 5.01 ± 0.49 mm, and thus, the first treatment started since the 8^th^ day. After all the treatments, the tumor volume in the APA-U group was significantly lower than that in the other groups (*P* < 0.05) (Table 1).  Figure 2 shows the tumor growth curve in different groups.

After the 14-day treatment, the tumors in the M group and UTMD group became significantly larger than those in the other two groups. The tumor growth in the APA group and APA-U group was inhibited to different extents. The tumor volume in the APA group increased slowly during the treatment; in the APA-U group, it increased slowly in the first week of treatment and then gradually decreased in the second week.

### 3.3. CEUS of Tumors

**Figure 3:**
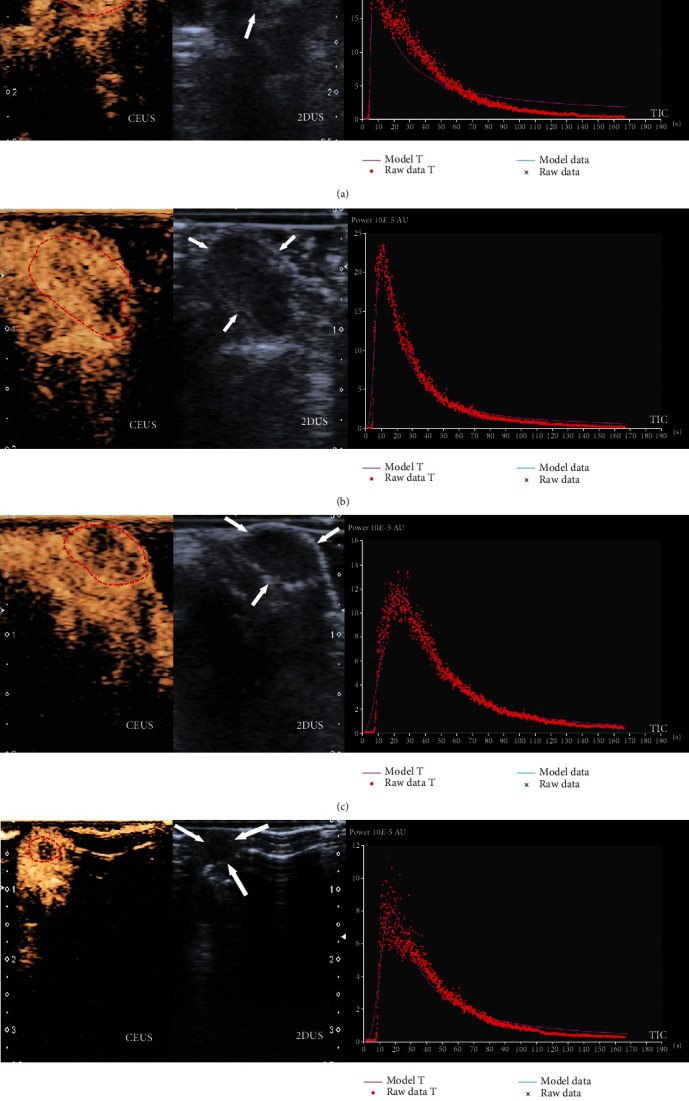
Tumors in different groups on CEUS. (a) An intense enhancement was shown in the tumor on CEUS (red circle) in the M group, and the tumor was heterogeneous hypoechoic on 2DUS and sized 10.8 mm × 10.0 mm × 9.8 mm (white arrow), with the PI of 25.1 (10*E*-5 AU) and the AUC of 858.9 (10*E*-5 AU·s) on TIC. (b) A heterogeneous enhancement with spotty filling defect was shown in the tumor on CEUS (red circle) in the UTMD group, and the tumor was heterogeneous hypoechoic on 2DUS and sized 11.2 mm × 10.9 mm × 6.6 mm (white arrow), with the PI of 22.5 (10*E*-5 AU) and the AUC of 724.3 (10*E*-5 AU·s) on TIC. (c) The heterogeneous enhancement with patchy filling defect was noted in the tumor on CEUS (red circle) in the APA group, and the tumor was homogeneous hypoechoic on 2DUS and sized 8.1 mm × 5.5 mm × 7.7 mm (white arrow), with the PI value of 11.6 (10*E*-5 AU) and the AUC of 589.9 (10*E*-5 AU·s) on TIC. (d) The heterogeneous enhancement with patchy filling defect and homogeneous hypoechoicity was observed in the tumor on CEUS (red circle) in the APA-U group, and the tumor had homogeneous hypoechoicity on 2DUS and a small size (6.9 mm × 6.0 mm × 5.1 mm; white arrow), with the PI of 8.0 (10*E*-5 AU) and the AUC of 372.1 (10*E*-5 AU·s) on TIC.

Compared with the M group, the CEUS parameters (PI and AUC) in the APA group and APA-U group decreased after the 14-day treatment, and the decrease in the APA-U group was more obvious than that in the APA group (*P* < 0.05), while there were no significant differences in both PI and AUC between the UTMD group and the M group (*P* > 0.05) (Table 2, Figure 3).

### 3.4. Blood Test Indicators

After the treatments, the levels of the WBC, PLT, and NE ratios in the APA and APA-U groups were all found lower than those in the M and UTMD groups (*P* < 0.05), while no significant difference of those levels was found between the APA group and the APA-U group (*P* > 0.05); meanwhile, there was no significant difference of both ALT and Scr levels found among the four different groups (*P* > 0.05) (Table 3).

### 3.5. VEGF Expression in Tumors

**Figure 4:**
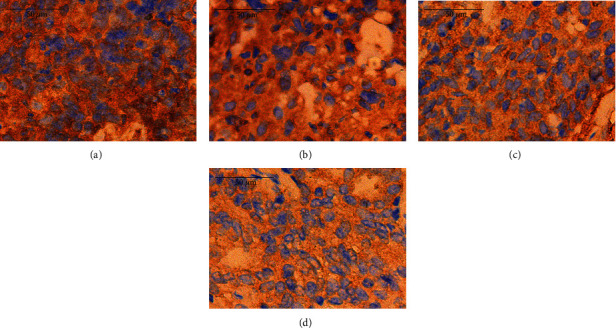
Immunohistochemistry for VEGF in different groups (×400). (a) A large number of brown granules were diffusely distributed in the cytoplasm of tumor cells in the M group, and the VEGF IOD was 8629.55. (b) A large number of brown granules were diffusely distributed in the cytoplasm of tumor cells in the UTMD group, and the VEGF IOD was 8045.29. (c) In the APA group, the brown flaky granules were distributed in the cytoplasm of tumor cells, and the VEGF IOD was 6986.39. (d) In the APA-U group, the brown granules were scattered in the cytoplasm of tumor cells, and the VEGF IOD was 5276.10.

**Figure 5:**
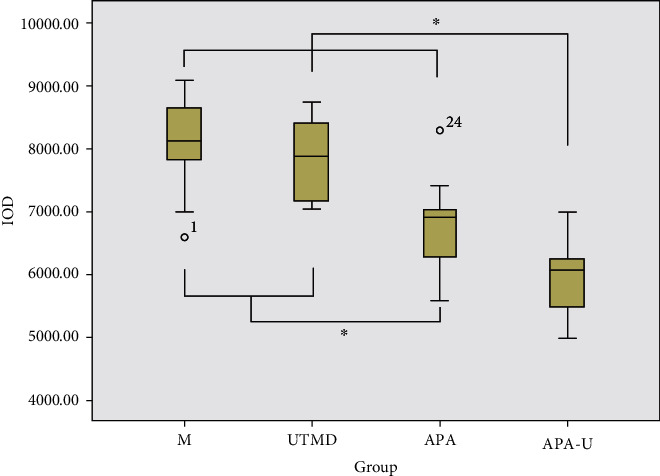
The VEGF IOD in different groups. Note: asterisks indicate the pairs having statistically significant differences in the ANOVA test followed by LSD *t*-test.

The VEGF protein was mainly expressed in the cytoplasm, and cells with brown granules in the cytoplasm were regarded as positive cells (Figure 4). After the treatments, the VEGF IOD in the APA group and the APA-U group was markedly lower than that in the M group and the UTMD group (*P* < 0.05), and the IOD in the APA-U group was the lowest among the four groups (*P* < 0.05) (Figure 5).

### 3.6. Neovascularization in Different Groups

**Figure 6:**
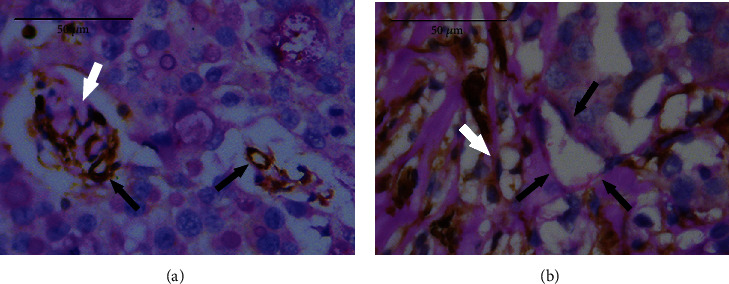
CD31-PAS double staining of tumor tissues after treatments (×400). (a) Brown granules (CD31-positive staining) were mainly observed in the endothelial cells under a light microscope, and PAS-positive staining was purple-red, showing coarse granular or fine mesh staining in the tumor cells and intercellular space. The IOD of CD31 and PAS was 286.53 and 281.79, respectively. The MV structures composed of endothelial cells with brown cytoplasm could be observed, showing different manifestations of endothelial cell cluster (white arrow) and vascular-like structure (black arrow). The IOD of CD31 and PAS of the MV structures was 183.56 and 5.06, respectively. (b) In a marginal area of the tumor, CD31-positive staining was noted in the endothelial cells, some tumor cells and intercellular space, and PAS-positive staining (purple-red) in the tumor cells and intercellular space showed coarse granular or fine mesh. The IOD of CD31 and PAS was 1601.93 and 1103.91, respectively. A mosaic blood vessel (black arrow) composed of endothelial cells and tumor cells was observed. In the structure, the endothelial cells were weakly positive for CD31, with the IOD of 9.26, and the cell matrix on the luminal surface showed PAS-positive staining, with the IOD of 31.99. White arrow: MV characterized by single endothelial cell.

**Figure 7:**
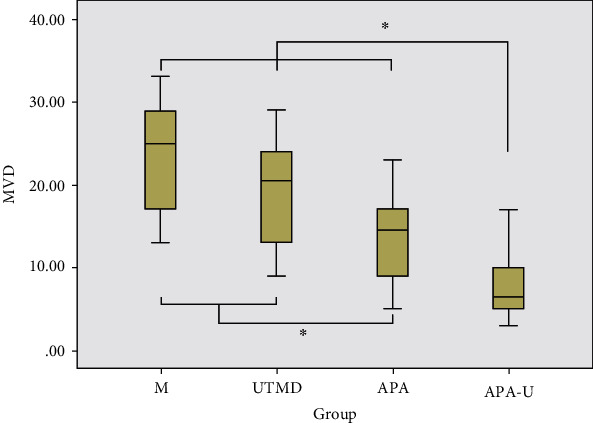
MVD in the tumor tissues after different treatments. Note: asterisks indicate the pairs having statistically significant differences in the ANOVA test followed by LSD *t*-test.

After CD31-PAS double staining, the CD31-positive cytoplasm of vascular endothelial cells showed brown granules (Figure 6(a)), and the PAS-positive staining could be observed in both tumor cells and intercellular space, showing the thick purple-red granules or fine mesh area. Clear VM structure was not found in the tumor tissues after treatments, but another heterogeneous vasculature-like mosaic blood vessel was observed (Figure 6(b)), which was composed of endothelial cells and tumor cells, with PAS-positive staining in the cell matrix on the luminal surface and weakly CD31-positive staining in the endothelial cells. In the present study, mosaic blood vessels were found only in 3 tumor tissues of the UTMD group, and the heterogeneous vascular positivity rate was 30% (3/10) in the UTMD group, but 0% (0/10) in other groups, while no significant difference was found in the heterogeneous vascular positivity rate among different groups (*P* > 0.0125). After the treatments, the MVD in the APA group and the APA-U group was markedly lower than that in the UTMD group and the M group (*P* < 0.05), and the APA-U group had the lowest MVD (*P* < 0.05) (Figure 7).

## 4. Discussion

Targeted antiangiogenic drugs exert an anticancer effect mainly by affecting the blood vessel formation in cancers, and their therapeutic efficacy has been confirmed in a variety of malignant tumors. However, studies about the antiangiogenic treatment of TNBC are still ongoing [17]. Small molecule tyrosine kinases inhibitor (TKI) is one of the most common anticancer drugs and can inhibit angiogenesis. Sorafenib and sunitinib are small molecule TKI with multiple targets and can exert an antiangiogenic effect by inhibiting the tyrosine kinase activity of some receptors such as VEGFR and platelet-derived growth factor receptor. However, they are not the recommended drugs for the treatment of TNBC. This may be related to the “off-target effect” of multitarget drugs in the treatment of cancers [18, 19]. In addition, the antiangiogenic drug may activate other pathways related to angiogenesis, stimulate the formation of heterogeneous vasculatures, and promote tumor metastasis [20]. Although APA is also a multitarget small molecule TKI, it highly targets VEGFR2 and has little influence on other receptors. The half inhibitory concentration of APA for VEGFR2 is much lower than that of sorafenib and sunitinib [21]. Thus, APA may be an ideal antiangiogenic drug. Our results showed that APA was able to inhibit the MV formation and suppress tumor growth of TNBC, which was consistent with previous findings [22]. However, APA can also act on VEGFR2 expressed in other tissues and organs as other antiangiogenic drugs, which may be responsible for some systemic side effects of APA in clinical practice, including hypertension, proteinuria, hand and foot skin reaction, thrombocytopenia, leukopenia, and neutropenia [23, 24].

The optimization of antitumor treatment is to minimize the toxic and side effects while ensuring the favorable antitumor effects. In the UTMD, a microbubble contrast agent at a certain dose is injected into the blood circulation, and then the tumor is irradiated with ultrasound to rupture the microbubbles entering the blood circulation of the tumor, causing a cavitation effect, which may damage the cell membrane and vascular endothelial cells. This will increase the vascular permeability and therefore the drug concentration increases in the tumor, leading to the improvement of drug bioavailability, the reduction of drug dose, and the decrease in the systemic side effects [14]. In addition, the cavitation effect can even cause the disintegration of microvasculature, local hematoma, and thrombosis, which blocks the blood supply to the tumor, reducing the blood perfusion [25, 26]. In the UTMD, the extent of the cavitation effect can be adjusted by changing the acoustic parameters, microbubble size, and microbubble concentration for ultrasound irradiation [27, 28]. In our previous study, irradiation of SonoVue microbubbles with low-frequency ultrasound at 0.75 W/cm^2^ for 2 min could temporarily block the blood perfusion to the TNBC xenograft tumors, and the blood perfusion was restored within 3 h after UTMD [29]. In the present study, the same ultrasound parameters were used. After UTMD for 14 consecutive days, the tumor growth was not significantly inhibited, and the MVD, CEUS parameters, VEGF expression, body weight, and blood parameters remained stable. These results suggested that the cavitation effect produced by UTMD with the present parameters had no effect on the blood perfusion, angiogenesis, and tumor growth of TNBC transplanted tumors, suggesting good safety. In our study, UTMD was used in combination with APA in the treatment of TNBC, which achieved a better antiangiogenic effect and tumor inhibitory effect as compared to APA alone. This was consistent with previous findings on UTMD [30, 31]. It may be related to the increased vascular permeability and cell membrane permeability due to the UTMD-induced cavitation, and thus, APA could easily enter the vascular endothelial cells and tumor cells.

In the present study, the nude mice in the APA-U group were in a stable general condition during the experiment, and there were no significant differences found between the APA-U group and the M group in body weight, weight change rate, and levels of liver and kidney function indexes of nude mice (*P* > 0.05), suggesting that the combined treatment with APA and UTMD had no obvious potential systemic toxicity. In this study, the WBC, PLT, and NE ratio levels of nude mice in the APA group and the APA-U group were lower than those in the M group (*P* < 0.05), but there was no difference between those in the UTMD group and the M group (*P* > 0.05), suggesting that the UTMD technology used in the experiment did not cause the decrease of WBCs, NEs and PLTs in nude mice. The systemic side effects of APA reported presently included leukopenia, neutropenia, and thrombocytopenia [23, 24]; therefore, the decrease of blood cell counts in the APA group and the APA-U group was considered as the side effect of APA. At the same time, there was no difference of the WBC, PLT, and NE ratio levels of nude mice found between the APA-U group and the APA group (*P* > 0.05), and there was no abnormal skin appearance in both groups. Therefore, we believed that the combined application of the UTMD technology did not significantly increase the severity of APA-related systemic side effects, such as leukopenia, neutropenia, thrombocytopenia, and hand-foot syndrome in the tumor-bearing nude mice. This may be explained as that, in the UTMD, the ultrasound irradiation focuses on the tumor, which only improves the permeability and APA concentration in the tumor, without affecting other parts of the body. In addition, the immunohistochemistry in the present study showed that the VEGF expression in the APA-U group was significantly lower than that in the other groups (*P* < 0.05), suggesting that the downregulation of VEGF expression may be one of the mechanisms underlying the inhibited angiogenesis in the TNBC after combined treatment with APA and UTMD.

The heterogeneous vasculature is a special microcirculation pattern in the tumor that is different from endothelial blood vessels, including VM and the mosaic blood vessel. The heterogeneous vasculature, together with MV, belongs to new blood vessels. Some investigators have proposed that VM refers to the tumor angiogenesis at the initial stage, which mainly occurs in the early stage of malignant tumors, and some factors such as hypoxia and VEGFR2 activation play key roles in the VM formation [30, 31]. VM is formed by tumor stem cells with VEGFR2 expression, and it is negative to CD31 and has no endothelial cells. The basement membrane of the VM lumen is composed of glycoprotein-rich substances secreted by tumor cells and is positive to PAS (purple-red) [15, 16]. The mosaic blood vessels are composed of tumor cells and endothelial cells and regarded as the intermediate stage between VM and major blood vessels. As the tumor grows, the VM gradually becomes a mosaic blood vessel and then is replaced with blood vessels composed of endothelial cells [15, 32]. The heterogeneous vasculature has no barrier function as endothelial cells, and therefore, tumors are susceptible to invasion and metastasis. The presence of heterogeneous vasculature increases the malignancy of tumors. Simple antiangiogenic therapy inhibits blood vessel formation alone and has no influence on the growth of tumor cells. Under this condition, the tumor cells may experience recoding to counteract with ischemia and hypoxia and then transform into tumor stem cells highly expressing VEGFR2, which is involved in the formation of heterogeneous vasculatures, reducing the antitumor effect of antiangiogenic drugs [33]. In the present study, the VM structure was not detected, and only three mosaic blood vessels were identified in the UTMD group. There was no difference in the heterogeneous vascular positivity rate among the four groups (*P* > 0.05). In addition, the combination of UTMD and APA achieved favorable efficacy in the treatment of TNBC: it not only significantly reduced the MV formation and VEGF expression, but also had no influence on the formation of heterogeneous vasculature. This may be related to the cavitation effect produced by UTMD, which promotes the APA to enter the cells and inhibit the tyrosine kinase activity of VEGFR2, thereby interfering with the formation of heterogeneous vasculatures by tumor stem cells expressing VEGFR2.

To date, many investigators have attempted to use chemically modified microbubbles which carry chemotherapeutic drugs in the UTMD, which may increase the target-delivery of chemotherapeutics and therefore elevate the local concentration of chemotherapeutics [34]. However, the stability of drug-loaded microbubbles should be improved, and the drug might be released before it reaches the target site. In addition, the development of drug-loaded microbubbles has a long way to go before their clinical application [14]. In the present study, APA was administered in a routine way, combined with UTMD, for the anti-TNBC therapy. This treatment was relatively simple and easy to implement. In summary, UTMD can enhance the inhibitory effect of APA on the angiogenesis and tumor growth of TNBC, and the combined application of UTMD and APA fails to significantly increase the formation of heterogeneous vasculatures and the severity of APA-related systemic side effects in the tumor-bearing nude mice. Thus, this treatment has certain clinical potential in the management of TNBC, but more studies are needed to confirm our findings.

## Figures and Tables

**Table 1:** Body weight and tumor volume before and after treatments in different groups (*n* = 10, $\bar{x}\pm \text{SD}$).

Group	Body weight (g)			Body weight change rate (%)	Tumor volume (mm^3^)	
	Before the inoculation (day 0)	Before the first treatment (day 8)	After the last treatment (day 22)		Before the first treatment (day 8)	After the last treatment (day 22)
M	20.39 ± 0.56	20.94 ± 0.52	20.08 ± 0.64	4.10 ± 2.36	63.31 ± 17.68	568.44 ± 105.49
UTMD	20.40 ± 0.49	20.77 ± 0.61	19.85 ± 0.71	4.41 ± 2.84	70.00 ± 19.99	450.69 ± 69.21
APA	20.44 ± 0.39	20.86 ± 0.59	19.64 ± 0.62	5.87 ± 2.76	68.30 ± 16.39	206.15 ± 68.96^∗^^#^
APA-U	20.31 ± 0.41	20.66 ± 0.60	19.55 ± 0.69	5.63 ± 2.74	64.61 ± 20.57	93.97 ± 39.72^∗^^#△^

Note: ^∗^*P* < 0.05 vs. M group; ^#^*P* < 0.05 vs. UTMD group; ^△^*P* < 0.05 vs. APA group.

**Table 2:** CEUS parameters in different groups (*n* = 10, $\bar{x}\pm \text{SD}$).

Group	PI (10*E*-5 AU)		AUC (10*E*-5 AU·s)	
	Before the first treatment (day 8)	After the last treatment (day 22)	Before the first treatment (day 8)	After the last treatment (day 22)
M	28.15 ± 8.55	26.17 ± 7.14	1088.33 ± 278.13	921.87 ± 197.45
UTMD	30.32 ± 7.23	23.46 ± 5.72	1046.54 ± 241.28	777.35 ± 166.34
APA	29.19 ± 6.16	15.37 ± 3.53^∗^^#^	1118.12 ± 312.26	597.27 ± 162.13^∗^^#^
APA-U	29.13 ± 8.05	9.93 ± 2.43^∗^^#△^	996.30 ± 201.44	440.35 ± 96.97^∗^^#△^

Note: ^∗^*P* < 0.05 vs. M group; ^#^*P* < 0.05 vs. UTMD group; ^△^*P* < 0.05 vs. APA group.

**Table 3:** Blood parameters after treatments in different groups (*n* = 10, $\bar{x}\pm \text{SD}$).

Group	WBC (×10^9^/l)	PLT (×10^9^/l)	NE (%)	ALT (U/l)	Scr (*μ*mol/l)
M	16.23 ± 3.16	1722.21 ± 352.78	15.98 ± 3.04	43.47 ± 7.44	27.07 ± 5.47
UTMD	16.58 ± 3.32	1643.77 ± 379.87	14.88 ± 3.33	38.58 ± 5.63	28.18 ± 5.75
APA	13.02 ± 2.83^∗^^#^	1394.82 ± 315.54^∗^	10.84 ± 2.40^∗^^#^	44.36 ± 6.22	32.46 ± 5.82
APA-U	12.17 ± 2.45^∗^^#^	1345.13 ± 323.32^∗^	10.08 ± 2.23^∗^^#^	43.26 ± 5.42	32.19 ± 5.43

Note: ^∗^*P* < 0.05 vs. M group; ^#^*P* < 0.05 vs. UTMD group.

## Data Availability

The data used to support the findings of this study are available from the corresponding author upon request.

## References

[1] Kumar P., Aggarwal R. (2016). An overview of triple-negative breast cancer. *Archives of Gynecology and Obstetrics*.

[2] Saravanan S., Vimalraj S., Pavani K., Nikarika R., Sumantran V. N. (2020). Intussusceptive angiogenesis as a key therapeutic target for cancer therapy. *Life Sciences*.

[3] Damaskos C., Garmpi A., Nikolettos K. (2019). Triple-negative breast cancer: the progress of targeted therapies and future tendencies. *Anticancer Research*.

[4] Jhan J. R., Andrechek E. R. (2017). Triple-negative breast cancer and the potential for targeted therapy. *Pharmacogenomics*.

[5] Linderholm B. K., Hellborg H., Johansson U. (2009). Significantly higher levels of vascular endothelial growth factor (VEGF) and shorter survival times for patients with primary operable triple-negative breast cancer. *Annals of Oncology*.

[6] Bell R., Brown J., Parmar M. (2017). Final efficacy and updated safety results of the randomized phase III BEATRICE trial evaluating adjuvant bevacizumab-containing therapy in triple-negative early breast cancer. *Annals of Oncology*.

[7] Bronte G., Andreis D., Bravaccini S. (2017). Sorafenib for the treatment of breast cancer. *Expert Opinion on Pharmacotherapy*.

[8] Lee A., Djamgoz M. B. A. (2018). Triple negative breast cancer: emerging therapeutic modalities and novel combination therapies. *Cancer Treatment Reviews*.

[9] Luu T., Frankel P., Chung C. (2014). Phase I/II trial of vinorelbine and sorafenib in metastatic breast cancer. *Clinical Breast Cancer*.

[10] Yardley D. A., Shipley D. L., Peacock N. W. (2015). Phase I/II trial of neoadjuvant sunitinib administered with weekly paclitaxel/carboplatin in patients with locally advanced triple-negative breast cancer. *Breast Cancer Research and Treatment*.

[11] Gao Z., Shi M., Wang Y., Chen J., Ou Y. (2019). Apatinib enhanced anti-tumor activity of cisplatin on triple-negative breast cancer through inhibition of VEGFR-2. *Pathology, Research and Practice*.

[12] Liu J., Liu Q., Li Y. (2020). Efficacy and safety of camrelizumab combined with apatinib in advanced triple-negative breast cancer: an open-label phase II trial. *Journal for ImmunoTherapy of Cancer*.

[13] Wu S., Zhang L., Li H., Xu J., Jiang C., Sun T. (2020). Combined use of apatinib mesylate and vinorelbine versus vinorelbine alone in recurrent or metastatic triple-negative breast cancer: study protocol for a randomized controlled clinical trial. *Trials*.

[14] Mullick Chowdhury S., Lee T., Willmann J. K. (2017). Ultrasound-guided drug delivery in cancer. *Ultrasonography*.

[15] Zhang S., Zhang D., Sun B. (2007). Vasculogenic mimicry: current status and future prospects. *Cancer Letters*.

[16] Yao X., Ping Y., Liu Y. (2013). Vascular endothelial growth factor receptor 2 (VEGFR-2) plays a key role in vasculogenic mimicry formation, neovascularization and tumor initiation by glioma stem-like cells. *PLoS One*.

[17] Pan T., Zhou D., Shi Z. (2020). Centromere protein U (CENPU) enhances angiogenesis in triple-negative breast cancer by inhibiting ubiquitin-proteasomal degradation of COX-2. *Cancer Letters*.

[18] Chung A. S., Kowanetz M., Wu X. (2012). Differential drug class-specific metastatic effects following treatment with a panel of angiogenesis inhibitors. *The Journal of Pathology*.

[19] Welti J. C., Powles T., Foo S. (2012). Contrasting effects of sunitinib within in vivo models of metastasis. *Angiogenesis*.

[20] Sun H., Zhang D., Yao Z. (2017). Anti-angiogenic treatment promotes triple-negative breast cancer invasion via vasculogenic mimicry. *Cancer Biology & Therapy*.

[21] Tian S., Quan H., Xie C. (2011). YN968D1 is a novel and selective inhibitor of vascular endothelial growth factor receptor-2 tyrosine kinase with potent activity in vitro and in vivo. *Cancer Science*.

[22] Ma Y., Yu J., Li Q., Su Q., Cao B. (2020). Addition of docosahexaenoic acid synergistically enhances the efficacy of apatinib for triple-negative breast cancer therapy. *Bioscience, Biotechnology, and Biochemistry*.

[23] Hu X., Zhang J., Xu B. (2014). Multicenter phase II study of apatinib, a novel VEGFR inhibitor in heavily pretreated patients with metastatic triple-negative breast cancer. *International Journal of Cancer*.

[24] Li Y. H., Zhou Y., Wang Y. W. (2018). Comparison of apatinib and capecitabine (Xeloda) with capecitabine (Xeloda) in advanced triple-negative breast cancer as third-line therapy: a retrospective study. *Medicine (Baltimore)*.

[25] Suzuki J., Ogawa M., Takayama K. (2010). Ultrasound-microbubble-mediated intercellular adhesion molecule-1 small interfering ribonucleic acid transfection attenuates neointimal formation after arterial injury in mice. *Journal of the American College of Cardiology*.

[26] Yang S., Wang P., Wang X., Su X., Liu Q. (2014). Activation of microbubbles by low-level therapeutic ultrasound enhances the antitumor effects of doxorubicin. *European Radiology*.

[27] Huang P., Zhang Y., Chen J. (2015). Enhanced antitumor efficacy of ultrasonic cavitation with up-sized microbubbles in pancreatic cancer. *Oncotarget*.

[28] Lu X., Dou C., Fabiilli M. L., Miller D. L. (2019). Capillary hemorrhage induced by contrast-enhanced diagnostic ultrasound in rat intestine. *Ultrasound in Medicine & Biology*.

[29] Jing Y., Xiu-Juan Z., Hong-Jiao C. (2019). Ultrasound-targeted microbubble destruction improved the antiangiogenic effect of Endostar in triple-negative breast carcinoma xenografts. *Journal of Cancer Research and Clinical Oncology*.

[30] Sasaki N., Kudo N., Nakamura K. (2012). Activation of Microbubbles by Short-Pulsed Ultrasound Enhances the Cytotoxic Effect of _Cis_ -Diamminedichloroplatinum (II) in a Canine Thyroid Adenocarcinoma Cell Line _In Vitro_. *Ultrasound in Medicine & Biology*.

[31] Dimcevski G., Kotopoulis S., Bjånes T. (2016). A human clinical trial using ultrasound and microbubbles to enhance gemcitabine treatment of inoperable pancreatic cancer. *Journal of Controlled Release*.

[32] di Tomaso E., Capen D., Haskell A. (2005). Mosaic tumor vessels: cellular basis and ultrastructure of focal regions lacking endothelial cell markers. *Cancer Research*.

[33] Mao Y., Zhu L., Huang Z. (2020). Stem-like tumor cells involved in heterogeneous vasculogenesis in breast cancer. *Endocrine-Related Cancer*.

[34] Wu Y., Sun T., Tang J., Liu Y., Li F. (2020). Ultrasound-targeted microbubble destruction enhances the antitumor efficacy of doxorubicin in a mouse hepatocellular carcinoma model. *Ultrasound in Medicine & Biology*.

